# Multifrequency electrical impedance tomography (Mf-EIT) for the detection of breast cancer phantom anomalies

**DOI:** 10.1016/j.mex.2024.103087

**Published:** 2024-12-06

**Authors:** Bayu Ariwanto, Khusnul Ain, Riries Rulaningtyas, Nuril Ukhrowiyah, Rohadatul Aisya, Ahmad Hoirul Basori, Andi Besse Fidausiah Mansur

**Affiliations:** a Magister of Biomedical Engineering, Faculty of Science and Technology, Airlangga University, Surabaya 60115, Indonesia; b Faculty of Computing and Information Technology in Rabigh, King Abdulaziz University, Rabigh 21911, Saudi Arabia

**Keywords:** Multifrequency, Electrical impedance tomography, Analog discovery, Breast cancer, Gauss-Newton, GREIT, *Multifrequency Electrical Impedance Tomography (Mf-EIT)*

## Abstract

Breast cancer is the most commonly diagnosed neoplasm and one of the most widespread cancers among women. The research advanced the Mf-EIT hardware through analogue discovery, component assessment, hardware integration, software creation, and data reconstruction utilizing Gauss-Newton and GREIT approaches. The breast cancer phantom consisted of a gelatin and sodium chloride solution. The position and number of anomalies in the reconstructed image correspond with the phantom. Anomalies in the reconstructed image are illustrated in red, indicating that they exhibit higher conductivity than their environment. The smallest percentage difference in conductivity between the reconstructed image of the cancer abnormality and the phantom is 0.18 %, recorded at a current of 0.35 mA and a frequency of 150 kHz. The smallest percentage difference in size between cancer abnormalities 1 and 2 in the reconstructed image and the phantom is 0.14 %, observed at a current of 0.22 mA and a frequency of 80 kHz. In brief,

• This study proposes an innovative Electrical Impedance Tomography (EIT)
• The designed and built the Mf-EIT hardware based on data reconstruction using Gauss-Newton and GREIT
• The Electrical Impedance Tomography designed to detect the anomalies in the reconstructed image of Breast Cancer.

## Graphical abstract

**Figure:**
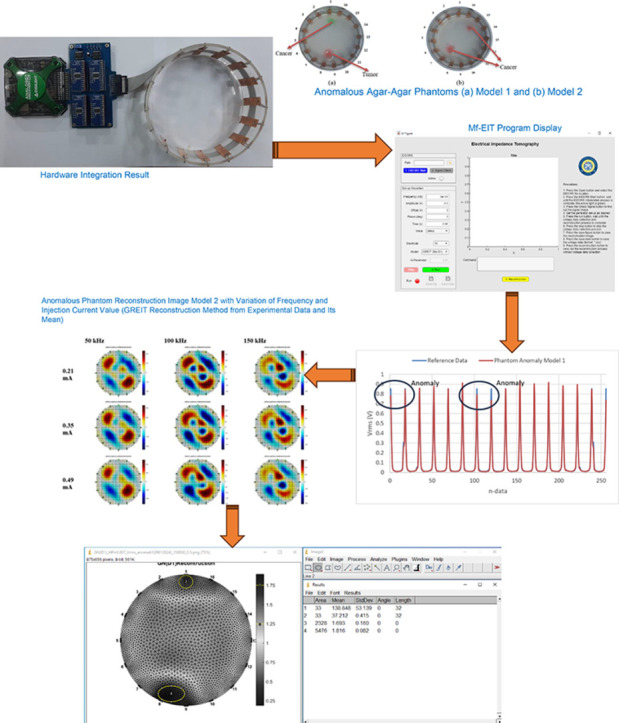

Specifications table

**Table:** 

Subject area:	Computer Science
More specific subject area:	*Biomedical*
Name of your method:	*Multifrequency Electrical Impedance Tomography (Mf-EIT)*
Name and reference of original method:	*NA*
Resource availability:	*MATLAB*

## Background

Breast cancer is the most common cancer diagnosed globally, after lung cancer. In 2020, an estimated 685,000 women died from breast cancer. By 2040, Arnold et al. estimate that the burden of breast cancer will rise to over 3 million cases and 1 million deaths annually. Delayed diagnosis of breast cancer can increase the likelihood of developing the disease at an advanced stage. The most frequent reasons for delaying diagnosis were lack of awareness of the cause of symptoms (41.5 %), low perceived severity (27.7 %), and fear of surgery (26.2 %) [1,[13], [14], [15], [16], [17], [18], [19]]. Based on research, breast cancer incidence rates vary widely by race and ethnicity. Although the exact cause of breast cancer remains unknown, several risk factors such as demographic, reproductive, hormonal, breast-related heredity, and lifestyle factors contribute to its incidence [2,[28], [29], [30]]. Early detection and rapid diagnosis are required to prevent high mortality in breast cancer patients. Existing screening and early diagnostic gold standards for breast cancer include mammography, magnetic resonance imaging (MRI), core needle biopsy (CNB), and ultrasonography (USG). The disadvantages of these techniques include their limited sensitivity for dense breast tissue, the potential for radiation side effects, the possibility of false positives and false negatives, the need for relatively expensive tools, and their reliance on skilled medical personnel, specifically radiology specialists [3]. Mammography has a slightly increased risk of breast cancer with increased x-ray exposure in women under 40 years of age [4]. The accuracy of an ultrasound is determined by the operator. Allergies to the contrast agent and the long time it takes make MRI unsuitable for early detection of breast cancer. CNB is suitable for high-risk cancer patients and is an invasive surgery [3,[20], [21], [22], [23], [24], [25], [26], [27]].

Based on these weaknesses, it is necessary to develop breast cancer detection that is portable, does not use ionizing radiation sources, is easy to operate, non-invasive, and can distinguish cancerous and normal tissues. Electrical Impedance Tomography (EIT) is an imaging technology that utilizes the conductivity properties of tissues and organs [[37], [38]]. Human tissues and organs have different conductivity distributions under certain physiological and pathological conditions [[31], [32], [33], [34], [35], [36]]. EIT has the advantages of being sensitive to changes in tissue function, non-invasiveness, and radiation-free. The medical world has widely utilized EIT for stroke detection, treatment evaluation of cerebral edema and mannitol dehydration, epileptic seizure monitoring, intraoperative monitoring of aortic arch replacement, and brain function imaging [[5], [6], [7], [8]].

Sapuan et al. (2017) conducted research on breast anomaly diagnosis using EIT. They constructed EIT using a microcontroller atmega 8535 for data acquisition and signal generator XR2206 [9]. Generator signals using XR2206 have disadvantages, including difficulty adjusting amplitude and frequency. The Atmega 8535 microcontroller has a sampling rate of 16 Ms/s, which is not very high. To overcome these limitations, this study proposes the development of a Multifrequency Electrical Impedance Tomography (Mf-EIT) system utilizing the Analog Discovery. Holland (2020) stated in his research that with higher sampling rate values (up to 100 Ms/s), better signal control, and compatibility with software such as MATLAB or Python, Analog Discovery provides a reliable platform for accurate data acquisition and multifrequency analysis.

The incorporation of multifrequency capability allows the system to exploit the distinct impedance properties of tissues at varying frequencies, enabling more precise differentiation between cancerous and normal tissues. Edson et al. (2020) state that Cancer tissues, known to exhibit higher conductivity compared to their surroundings, become more distinguishable at specific frequencies, thus improving the accuracy of reconstructed images. This advancement is particularly important in the context of breast cancer diagnosis, where early detection relies on the ability to identify anomalies with high sensitivity. By addressing the limitations of previous systems and leveraging multifrequency imaging, the proposed Mf-EIT system represents a significant step forward in the development of non-invasive and radiation-free diagnostic technologies.

## Method details

EIT utilizing red pitaya can detect bone fractures. This study utilized a bone phantom composed of polylactic acid (PLA) alongside a human femur bone model fabricated via 3D printing technology. Reconstruction utilizing EIDORS. The reconstruction results indicate that red-pitaya-based EIT can differentiate between normal and broken bones [10]. The red-pitaya-based EIT has drawbacks, including the necessity for an external power supply, a protracted data retrieval process, and dependence on a Wi-Fi network for connectivity to the red-pitaya module.

In light of these issues, researchers advocate for the creation of an EIT system incorporating an Analog Discovery module, which features a 2-channel signal generator, a 2-channel oscilloscope, a power supply, 16 digital input/output ports, and a timer. This module boasts a substantial sample rate of 100 Ms/s, a 14-bit ADC, and the capability to measure signals with frequencies up to 20 MHz. All components can be managed using Waveform, Matlab, or Python software. In this investigation, researchers employed multifrequency alternating current (AC) injection and analyzed the resultant phase difference. Roh et al. (2023) assert that multifrequency impedance serves as a dependable method for evaluating the severity of lymphedema following breast cancer surgery. The principle of multifrequency bioimpedance posits that each tissue exhibits a distinct impedance at a certain frequency [11]. Multifrequency EIT is anticipated to enhance the quality and precision of breast cancer imaging.

This research encompasses hardware design, software design, breast phantom construction, and analysis. The MATLAB program facilitates data collecting by managing the signal generator, oscilloscope, power supply, and digital port of the Analogue Discovery module, whereas EIDORS is employed to reconstruct voltage data into pictures via the Gauss-Newton and GREIT methodologies. The test subject is a breast phantom composed of agar and NaCl solution, with image analysis conducted using ImageJ.

**Fig. 1:**
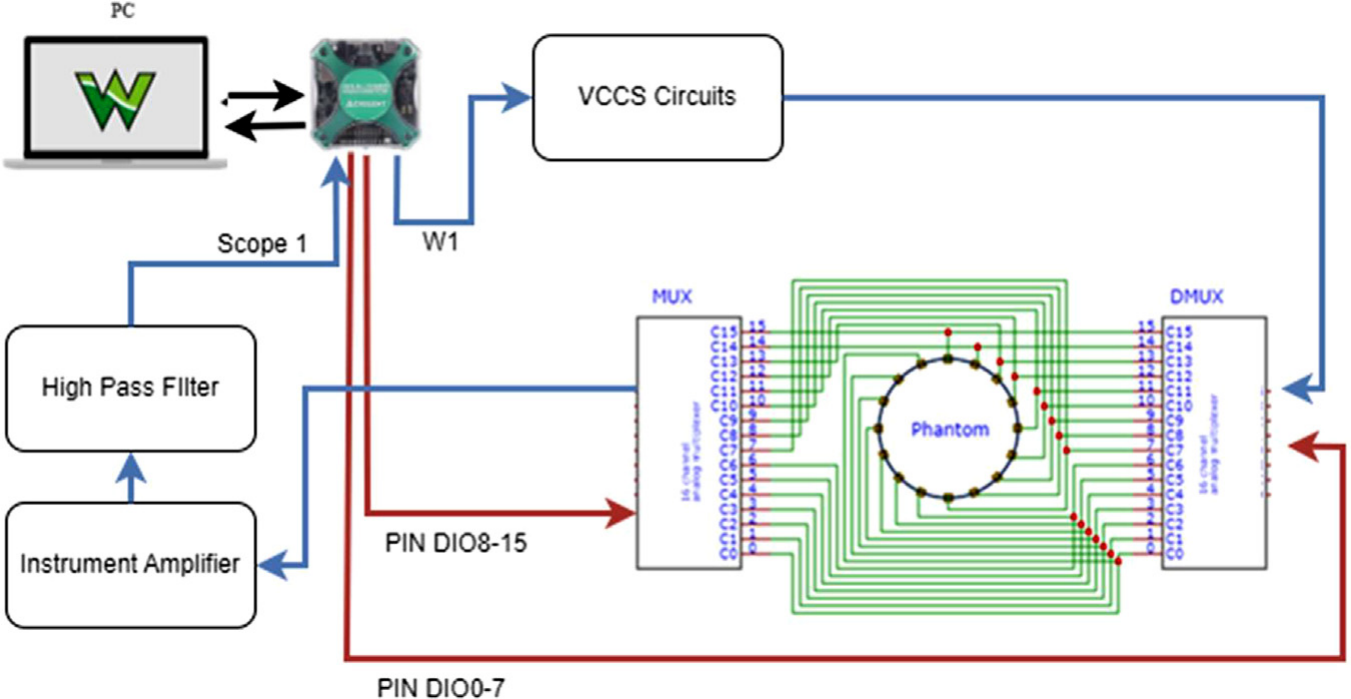
Block Diagram of Mf-EIT Design based on Analog Discovery.

The hardware design comprises a voltage-controlled current source (VCCS), an instrumentation amplifier, a multiplexer-demultiplexer (MUX-DMUX), and a direct current (DC) block. The block diagram in Fig. 1 illustrates the Analogue Discovery-based Mf-EIT for breast cancer diagnosis, while Fig. 6 presents the flowchart of the MATLAB software that governs it.

**Fig. 2:**
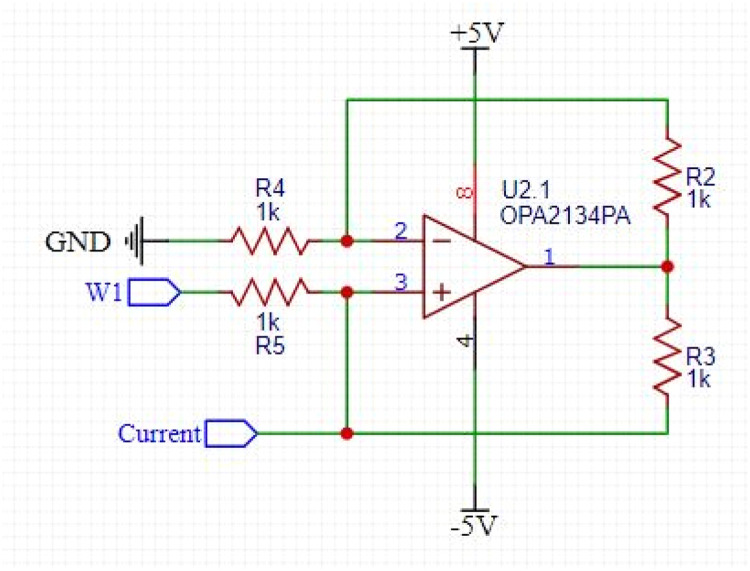
VCCS Circuit Schematic.

The generator signal from Analogue Discovery was evaluated to confirm its proper functionality and stability within a frequency range of 1 kHz to 1 MHz. The VCCS design adheres to the schematic depicted in Fig. 2, employing an OPA2134 and four 1 kΩ resistors. The OPA2134 offers an 8 MHz bandwidth, minimal distortion, high speed, and low noise levels.

**Fig. 3:**
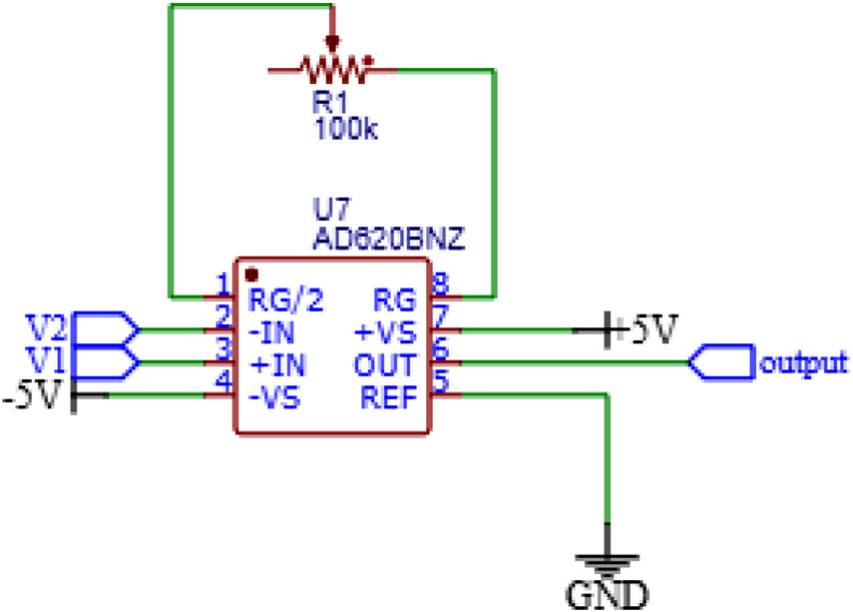
Instrumentation Amplifier Circuit Schematic.

The IA circuit uses IC AD620 with a variable resistor of 100 kΩ as the gain in Fig. 3. The input voltages V1 and V2 are connected to the MUX while the output voltage is connected to the DC block circuit.

**Fig. 4:**
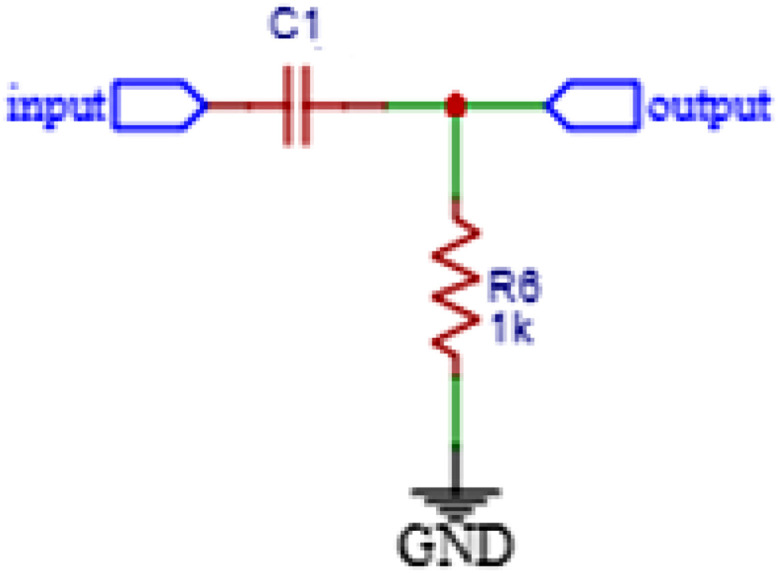
Block DC Schematic.

The DC block uses a high pass filter (HPF) circuit with a 1 µF capacitor, 1 kΩ resistor in Fig. 4. This circuit is used to provide a 0 V offset in the AC voltage.

**Fig. 5:**
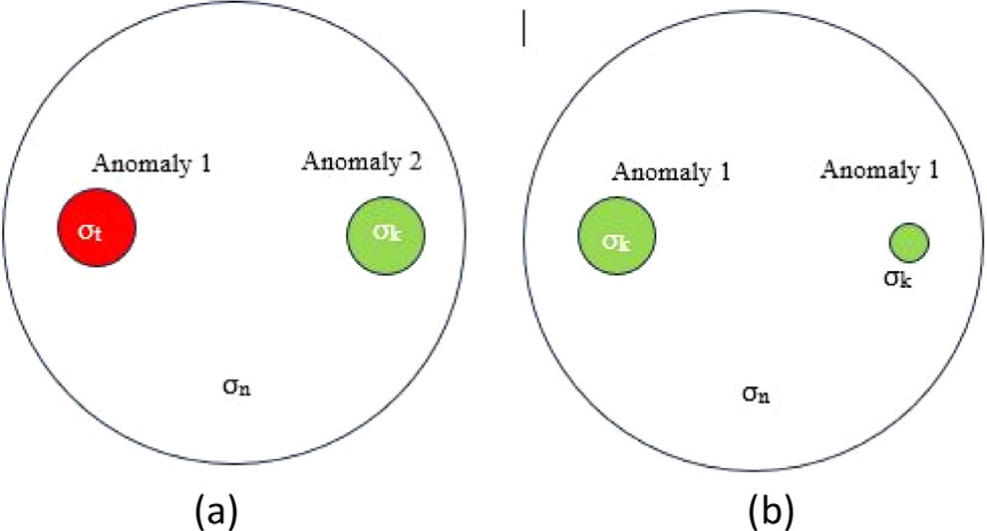
Anomalous Structure on Anomalous Phantoms a) Model 1, b) Model 2 Description: σ_t_ = Tumor Tissue conductivity (4.28 mS/cm), σ_k_ = conductivity of Cancer Tissue (1.71 mS/cm), σ_n_ = Normal Tissue conductivity (0.22 mS/cm).

**Fig. 6:**
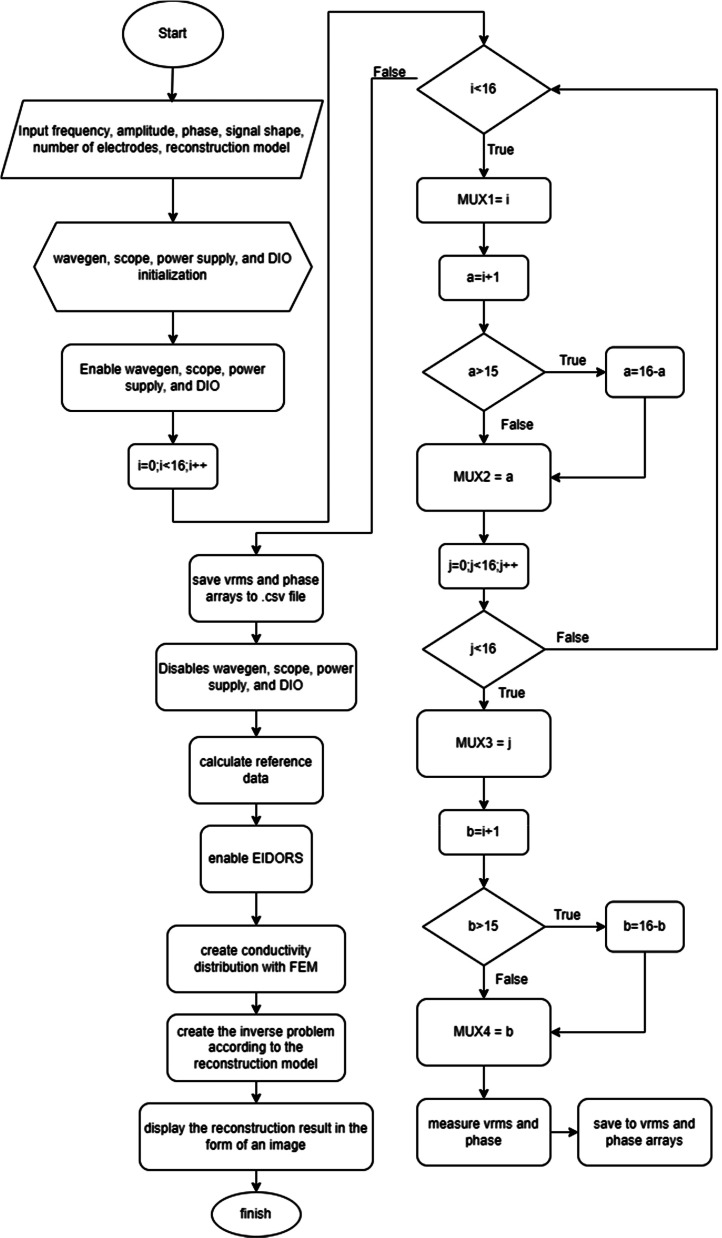
Flowchart of EIT 16 Electrode Program.

Breast phantoms are composed of agar and NaCl; an increase in NaCl concentration results in decreased resistivity and increased conductivity [12]. The correlation between NaCl solution concentration and conductivity is described by equation (2.39) and presented in Table 1. This study developed two phantom models, each containing two anomalies. Model 1 features a cancer anomaly of varying sizes, while Model 2 includes both a cancer and a tumor anomaly of identical size, as illustrated in Fig. 5. Voltage data were acquired using the adjacent method with an injection current ranging from 0.21 mA to 0.56 mA and a frequency spanning from 50 kHz to 150 kHz. (Fig. 6)

**Table 1:** Relationship between NaCl Solution Concentration and Tissue Conductivity Value.

Conductivity Value	NaCl Solution Concertration
Normal Tissue Conductivity (0.22 mS/cm)	0.11 g/L
Conductivity of Breast Cancer Tissue (1.71 mS/cm)	0.85 g/L
Tumor Tissue Conductivity (center) (4.28 mS/cm)	2.14 g/L

The analysis was conducted visually by comparing the reconstructed images from the Gauss-Newton and GREIT methods with the phantom anomaly, including its position, size, and conductivity.

### Method validation

**Fig. 7:**
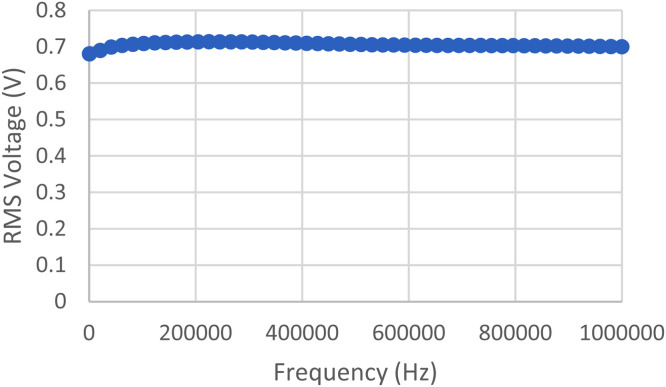
Analog Generator Discovery Test Result Graph.

The Analogue Discovery Signal Generator can create a consistent rms (root mean square) voltage of 0.7 Volts in a frequency range of 1 kHz to 1 MHz, as is displayed in Fig. 7.

**Fig. 8:**
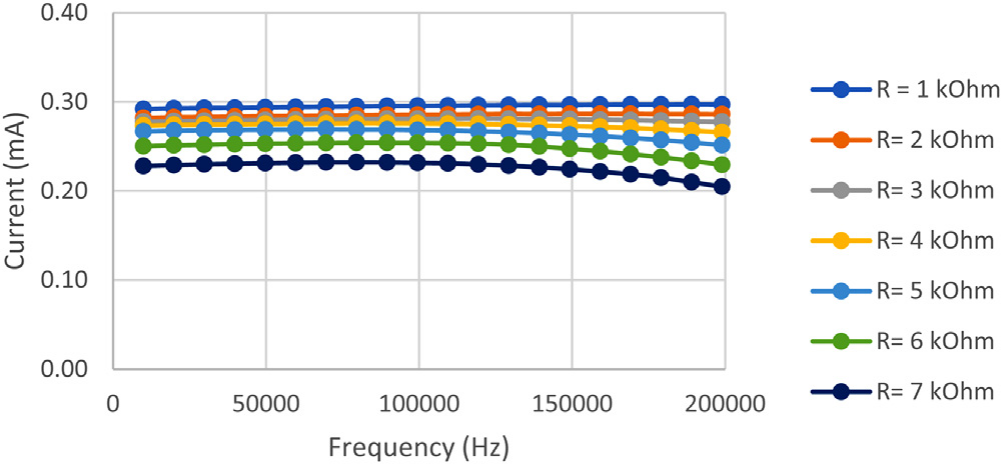
VCCS Circuit Test Result Graph.

The VCCS can generate a stable current at a load of 1 kΩ to 7 kΩ within the frequency range of 10 kHz to 200 kHz, as illustrated in Fig. 8. The fluctuation of the injection current remains within safe parameters for the body, as it is below 1 mA.

**Fig. 9:**
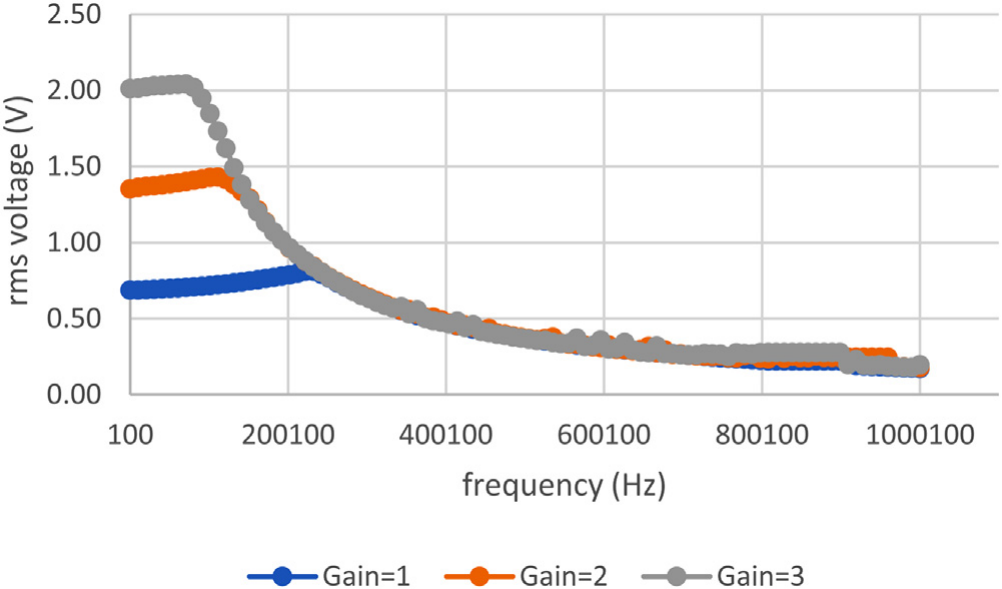
Instrument Amplifier (IA) Test Result Graph.

**Fig. 10:**
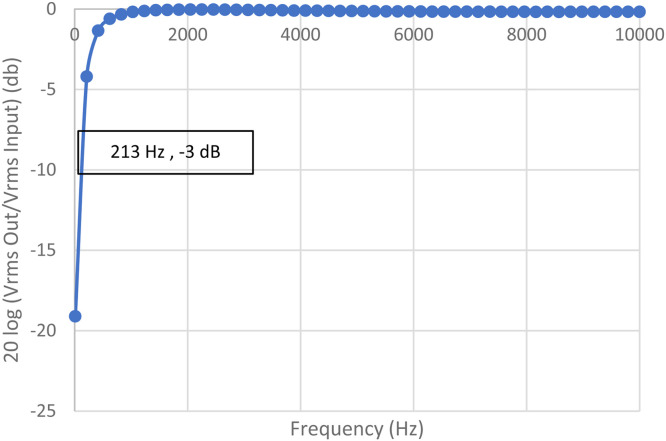
Block Circuit Test Result Graph.

The Instrument Amplifier test was conducted by measuring the rms voltage at various frequencies, resulting in a graph as shown in Fig. 9*.* The DC block can produce the frequency cut-off below 213 Hz as shown in Fig. 10.

**Fig. 11:**
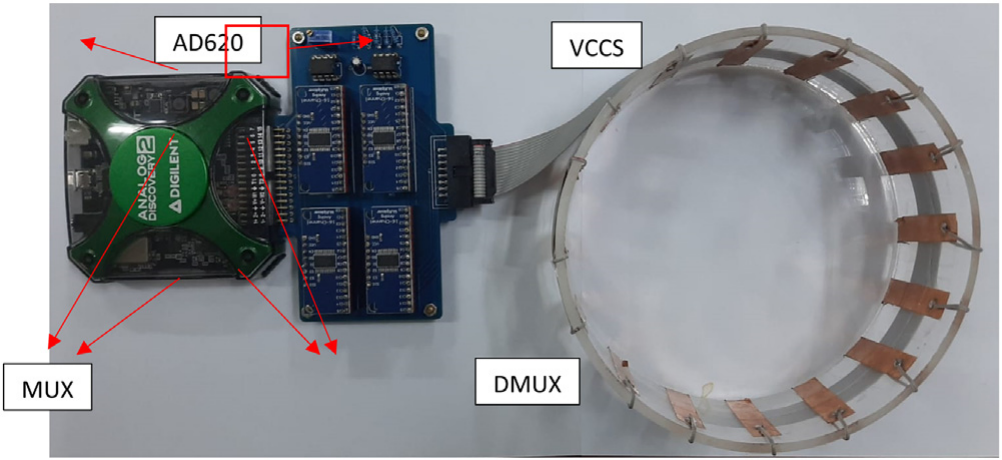
Hardware Integration Result.

**Fig. 12:**
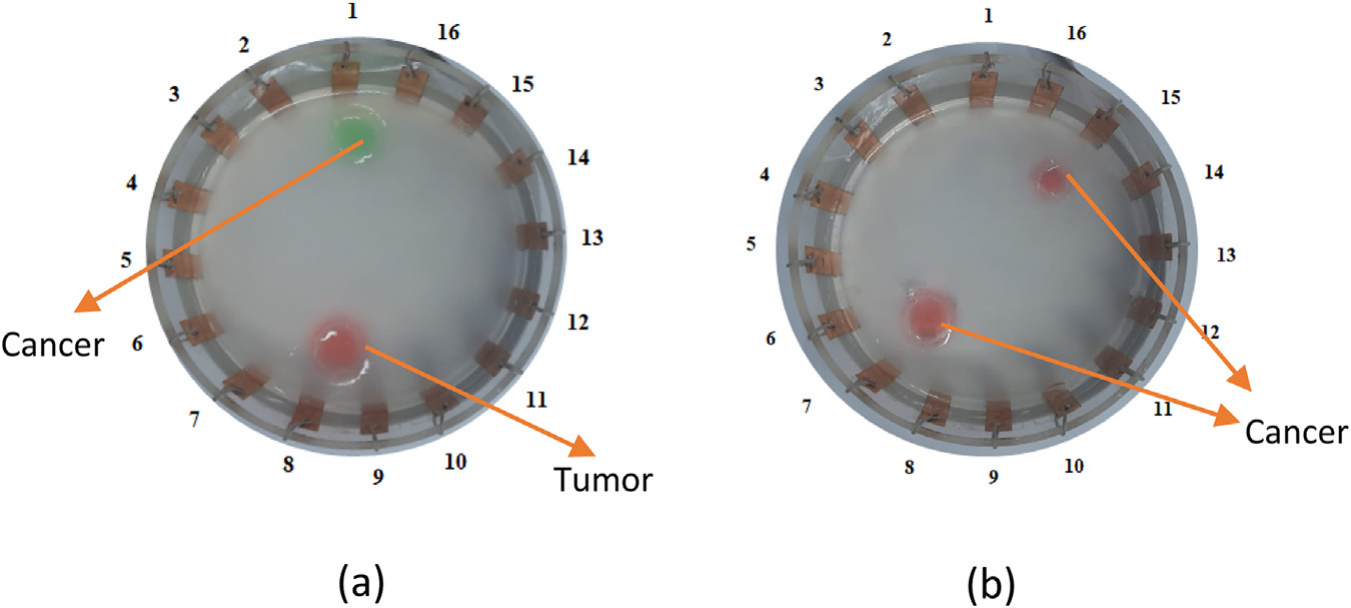
Anomalous Gelatine Phantoms (a) Model 1 and (b) Model 2.

The hardware integration resulted in Fig. 11 with Mf-EIT dimensions of 16 cm x 11 cm, while the difference phantom shown in Fig. 12.

**Fig. 13:**
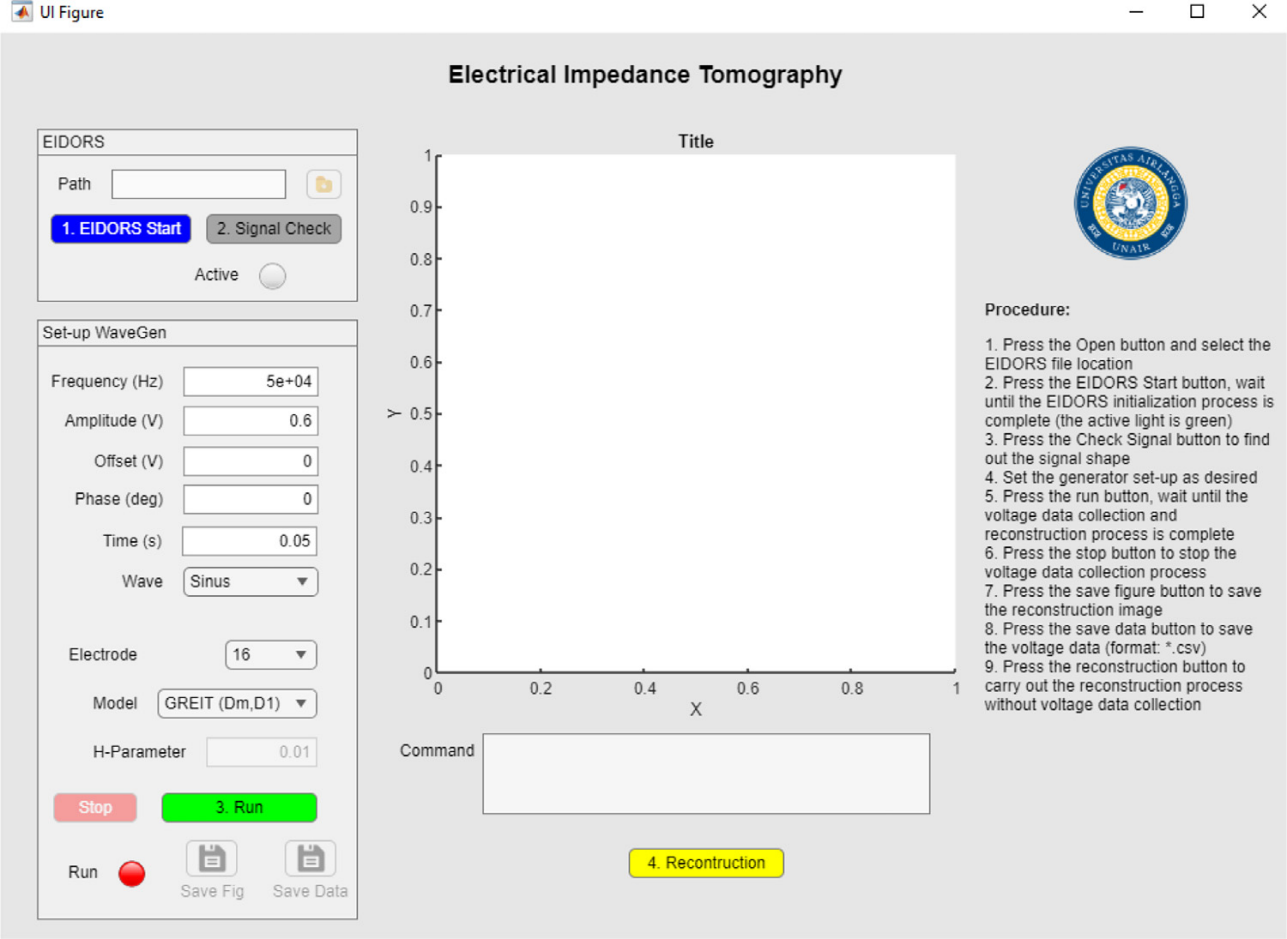
Mf-EIT Program Display.

Fig. 13 illustrates the Matlab software employed to manage the signal generator, oscilloscope, power supply, and DIO pins utilizing the adjacent method for data acquisition. The output voltage is recorded using an oscilloscope and stored in a CSV (comma-separated values) file format.

**Fig. 14:**
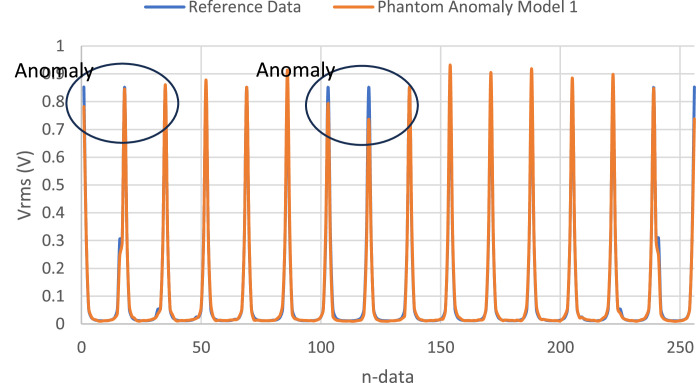
Graph of Model 1 Anomalous Voltage Measurement and Average Data at 50 kHz Frequency and Current Value of 0.21 mA.

Data were obtained at currents of 0.21 mA, 0.35 mA, and 0.49 mA, with frequency variations of 50 kHz, 100 kHz, and 150 kHz. The reference data is derived from the average measurements of the anomalous phantom. A graph depicting the voltage readings on the anomalous phantom model 1 alongside the reference using the average data is presented in Fig. 14.

**Fig. 15:**
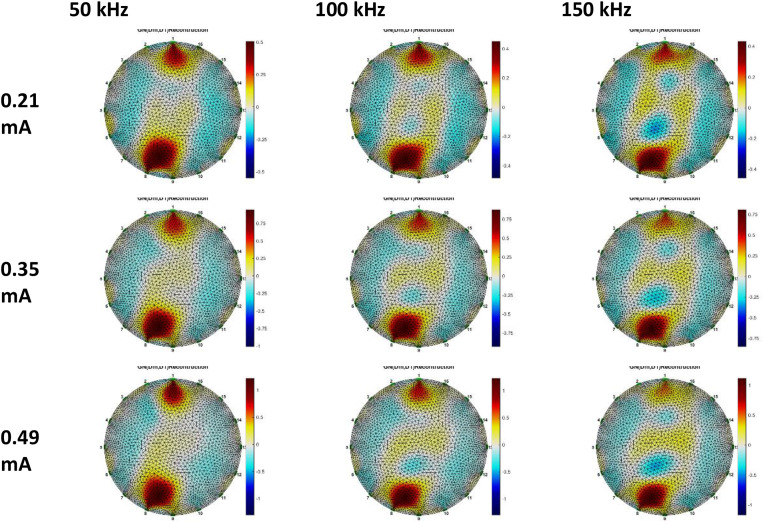
Anomalous Phantom Reconstruction Image Model 1 with Variation of Frequency and Injection Current Value (Gauss-Newton Reconstruction Method relative to experimental data and its mean).

**Fig. 16:**
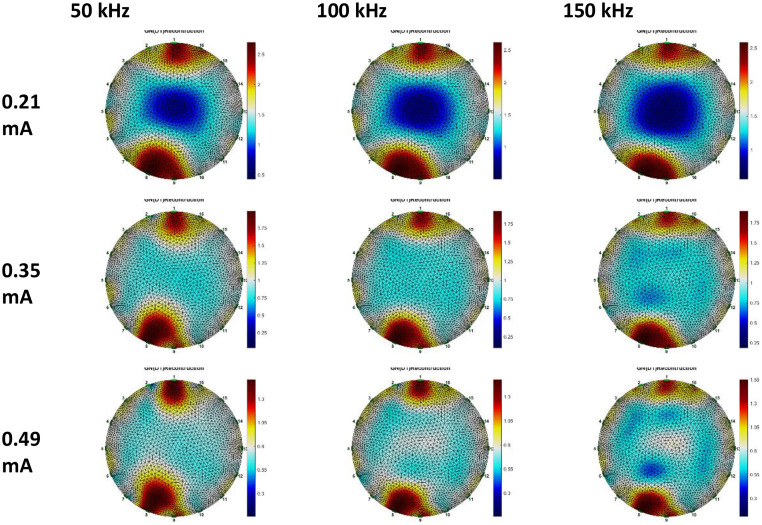
Anomalous Phantom Reconstruction Image Model 1 with Variation of Frequency and Injection Current Value (Absolute Gauss-Newton Reconstruction Method from experimental data.

**Fig. 17:**
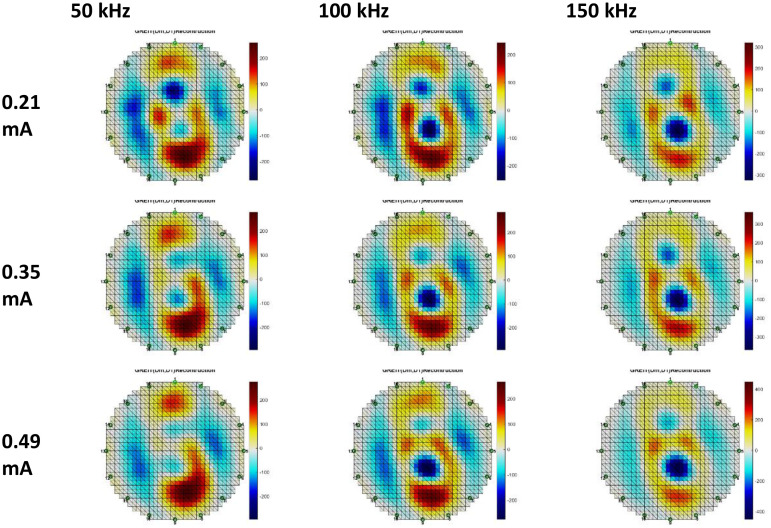
Anomalous Phantom Reconstruction Image Model 1 with Variation of Frequency and Injection Current Value (GREIT Reconstruction Method from experimental data and its mean).

**Fig. 18:**
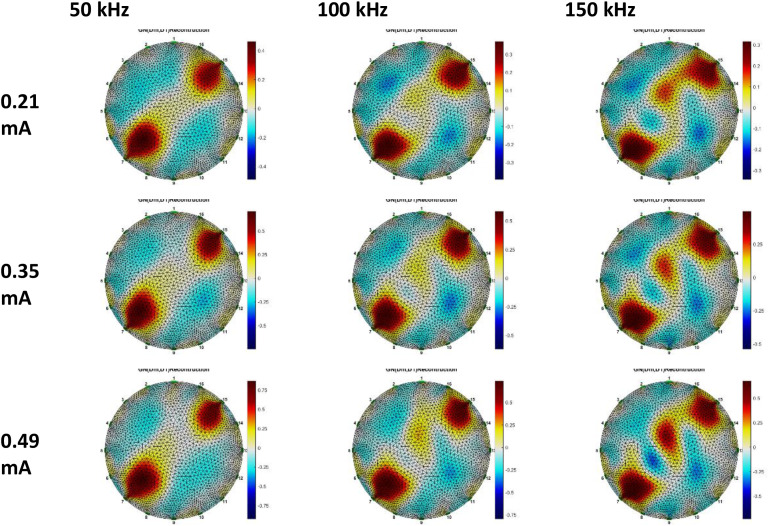
Anomalous Phantom Reconstruction Image Model 2 with Variation of Frequency and Injection Current Value (Gauss-Newton Reconstruction Method relative to experimental data and its mean).

**Fig. 19:**
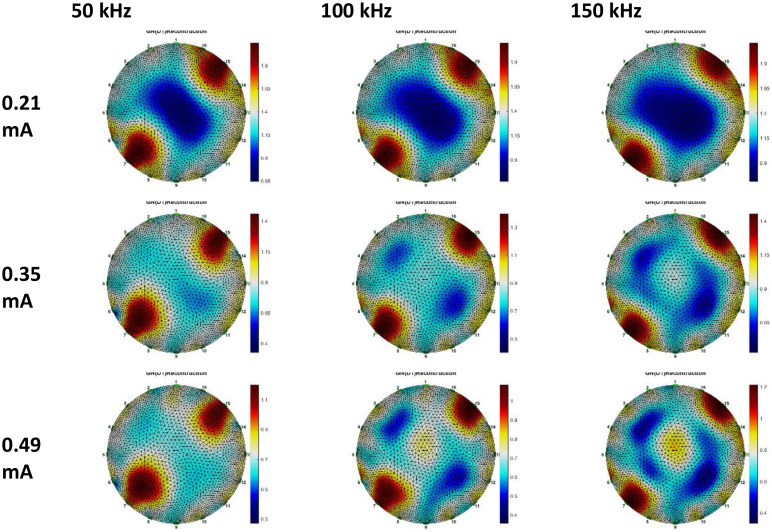
Anomalous Phantom Reconstruction Image Model 2 with Variation of Frequency and Injection Current Value (Gauss-Newton Reconstruction Method from experimental data).

**Fig. 20:**
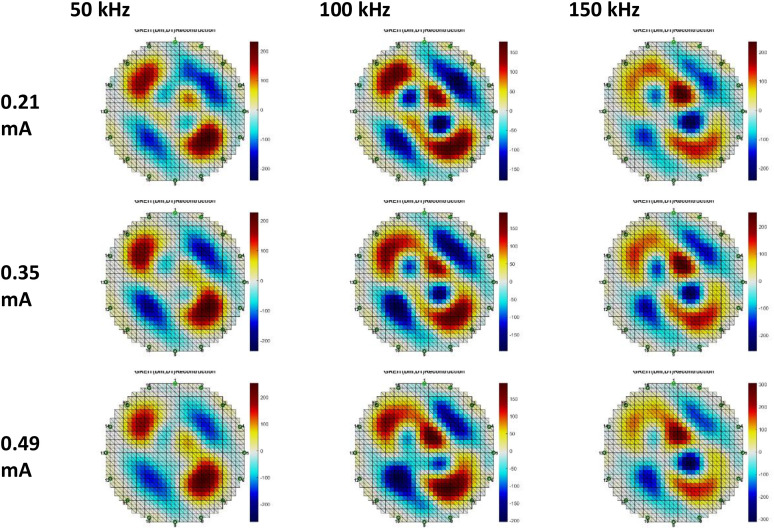
Anomalous Phantom Reconstruction Image Model 2 with Variation of Frequency and Injection Current Value (GREIT Reconstruction Method from Experimental Data and Its Mean).

Breast phantom voltage data, reconstructed using Gauss-Newton and GREIT methodologies. Three datasets were utilized for the GN method reconstruction: anomalous phantom data compared to homogeneous phantom data, anomalous phantom data compared to average data, and anomalous phantom voltage data exclusively. Fig. 15, Fig. 16, Fig. 17, Fig. 18, Fig. 19, Fig. 20 display the reconstructed images of anomalous phantoms 1 and 2, utilizing different frequency and current values.

**Fig. 21:**
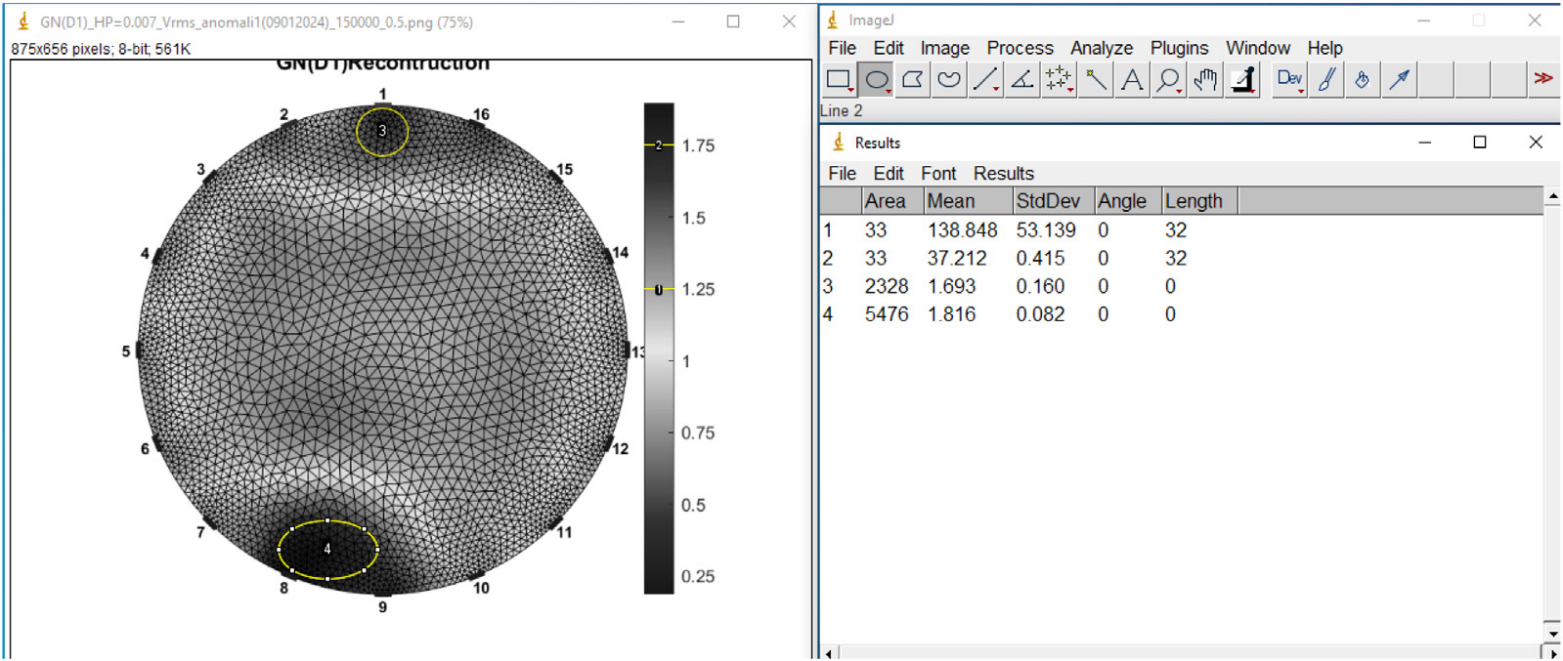
Calculation of Conductivity of Reconstructed Anomaly Image with Image J Program.

Quantitative analysis can be performed by assessing the conductivity value of the reconstructed image anomaly utilizing the Image J program. The measurement of the conductivity of the reconstructed image anomaly is illustrated in Fig. 21, while the conductivity values are presented in Table 2, Table 3.

**Table 2:** Conductivity Value of Cancer Anomaly in Reconstructed Image.

Current (mA)	50	100	150
**0.21**	2.48	2.49	2.46
**0.35**	1.74	1.73	1.71
**0.49**	1.34	1.31	1.34

**Table 3:** Conductivity Value of Tumor Anomaly in Reconstructed Image.

Current (mA)	50	100	150
**0.21**	2.55	2.55	2.50
**0.35**	1.82	1.78	1.77
**0.49**	1.35	1.34	1.38

The % difference in conductivity values is calculated using Eq. (1).

(1) $$\%\;different=\left|\frac{\sigma_{c}-\sigma_{f}}{\sigma_{f}}\right|x\;100\%$$

In this context, σ_c represents the image anomaly conductivity, while σ_f denotes the phantom anomaly conductivity. The percentage difference in conductivity values is exclusively assessed on the reconstructed image of the GN model, utilizing stress data sourced solely from the anomalous phantom (absolute measurement). This adjustment acknowledges the impracticality of acquiring homogeneous voltage data from the breast. The calculation of the percentage difference in conductivity values was performed on the reconstructed image of anomaly phantom model 1, which possesses identical anomaly dimensions but varying conductivity values. The tables detailing the percentage differences in conductivity values between cancer and tumor anomalies in the reconstructed image with the phantom are presented in Table 4, Table 5. Additionally, Table 6 illustrates the average percentage of conductivity difference between cancer and tumor anomalies in the reconstructed image.

**Table 4:** Percentage value of conductivity difference of cancer anomaly in reconstructed image with phantom.

Current (mA)	50	100	150
**0.21**	44.74	45.73	43.74
**0.35**	1.46	0.94	0.18
**0.49**	21.70	23.33	21.93

**Table 5:** Percentage value of conductivity difference of tumor anomaly in reconstructed image with phantom.

Current (mA)	50	100	150
**0.21**	40.49	40.49	41.66
**0.35**	57.57	58.34	58.57
**0.49**	68.43	68.62	67.76

**Table 6:** Area of Cancer Anomaly Model 1.

Current (mA)	50	100	150	
0.21	2.30	3.15	2.52	0.79
0.35	3.09	2.84	1.43	0.79
0.49	4.09	4.11	3.24	0.79

**Fig. 22:**
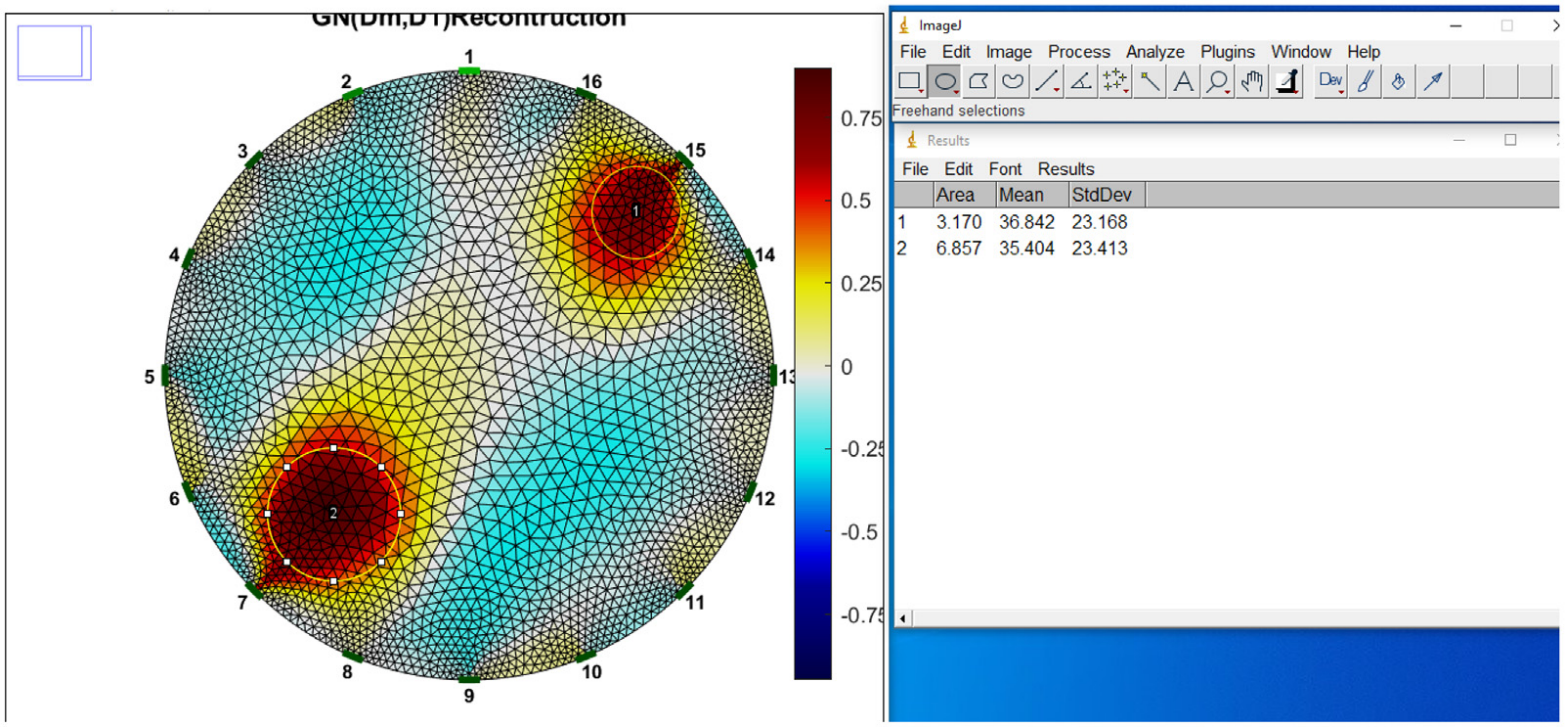
Measurement of Anomaly Area in Reconstructed Image Using Image J Program.

The next quantitative analysis was to measure the anomaly area on the reconstructed image and compare it with the anomaly area on the phantom. Area measurement was performed using the Image J program. Prior to measurement, area calibration was performed by calculating the area per pixel. As a reference, the area of a phantom with a diameter of 13.5 cm was used. The measurement of the anomalous area is shown in Fig. 22. This analysis is only applied to the reconstructed image of the GN method with the stress data source of the anomalous phantom model 2 only. Anomaly phantom model 2 is a phantom that has two anomalies with the same conductivity value of 1.71 mS/cm (cancer tissue conductivity) but different sizes, namely 2 cm and 1 cm in diameter. Next, the comparison of the tumor anomaly area in the reconstructed image with the cancer anomaly area in the reconstructed image is calculated. The results of these calculations need to be multiplied by a multiplying factor of 2. The results of the area of cancer anomalies 1 and 2 in the reconstructed image are shown in Table 6, Table 7. The results of the percentage difference in comparison of cancer anomalies 2 with 1 on the reconstructed image to the phantom are shown in Table 8.

**Table 7:** Area of Cancer Anomaly Model 2.

Current (mA)	50	100	150	
0.21	3.30	4.74	3.39	3.14
0.35	5.92	4.70	2.52	3.14
0.49	6.96	5.45	2.47	3.14

**Table 8:** Percentage difference in area ratio of cancer anatomical area of model 2 and 1 in reconstructed image with phantom.

Current (mA)	50	100	150
0.21	28.19	24.67	32.68
0.35	4.28	17.10	11.74
0.49	14.90	33.70	61.98

Table 6 shows the quantitative analysis of the percentage difference in area between the area of cancer anomalies in Model 1 and Model 2. The measurement was performed with ImageJ after area per pixel calibration. This difference provides insight into the accuracy of the system in reconstructing anomalies of different sizes on the two phantom models

A practical and portable Multifrequency Electrical Impedance Tomography (EIT) device based on Analog Discovery with MATLAB as the data acquisition program has been successfully developed. The device can generate currents ranging from 0.2 mA to 0.5 mA and operate within a frequency range of 50 kHz to 150 kHz for detecting anomalies in breast cancer models made of agar and NaCl solution. Visually, the anomaly images produced by the Gauss-Newton reconstruction method are superior (with better separation, lower noise, and a shape more similar to the anomaly in the phantom) compared to the GREIT method on this Analog Discovery-based Multifrequency Electrical Impedance Tomography (Mf-EIT) device. However, the reconstruction process using the GREIT method is faster than the Gauss-Newton method.

## Limitations

None.

## Ethics statements

Ethics approval is not required for this type of study.

## CRediT authorship contribution statement

**Bayu Ariwanto:** Conceptualization, Writing – original draft, Writing – review & editing. **Khusnul Ain:** Funding acquisition, Methodology, Resources, Conceptualization, Writing – original draft, Writing – review & editing. **Riries Rulaningtyas:** Data curation, Writing – original draft. **Nuril Ukhrowiyah:** Formal analysis, Writing – original draft, Writing – review & editing. **Rohadatul Aisya:** Formal analysis, Resources, Writing – original draft. **Ahmad Hoirul Basori:** Funding acquisition, Resources, Writing – review & editing. **Andi Besse Fidausiah Mansur:** Funding acquisition, Methodology, Writing – review & editing.

## Declaration of competing interest

The authors declare that they have no known competing financial interests or personal relationships that could have appeared to influence the work reported in this paper.

## References

[1] Hutajulu S.H., Prabandari Y.S., Bintoro B.S., Wiranata J.A., Widiastuti M., Suryani N.D., Saptari R.G., Taroeno-Hariadi K.W., Kurnianda J., Purwanto I., Hardianti M.S., Allsop M.J. (2022). Delays in the presentation and diagnosis of women with breast cancer in Yogyakarta. Indonesia: A retrospective observational study. PLoS. One.

[2] Momenimovahed Z., Salehiniya H. (2019). Breast Cancer: Targets and Therapy.

[3] Sornambikai S., Divya K.P., Vasanth S., Viswanathan C., Ponpandian N. (2023). CRISPR based biosensing: An ultrasensitive theranostic tool for the detection of early Breast Cancer biomarkers – A mini review. Biosensors and Bioelectronics: X.

[4] Du Y., Xie F., Yin L., Yang Y., Yang H., Wu G., Wang< S. (2022). Breast cancer early detection by using Fourier-transform infrared spectroscopy combined with different classification algorithms. Spectrochimica Acta - Part A: Molecular and Biomolecular Spectroscopy.

[5] Hannan S., Faulkner M., Aristovich K., Avery J., Walker M.C., Holder D.S. (2020). In vivo imaging of deep neural activity from the cortical surface during hippocampal epileptiform events in the rat brain using electrical impedance tomography. Neuroimage.

[6] Li Y., Zhang D., Liu B., Jin Z., Duan W., Dong X., Fu F., Yu S., Shi X. (2018). Noninvasive Cerebral Imaging and Monitoring Using Electrical Impedance Tomography During Total Aortic Arch Replacement. J. Cardiothorac. Vasc. Anesth..

[7] Witkowska-Wrobel A., Aristovich K., Crawford A., Perkins J.D., Holder D. (2021). Imaging of focal seizures with Electrical Impedance Tomography and depth electrodes in real time. Neuroimage.

[8] Yang B., Li B., Xu C., Hu S., Dai M., Xia J., Luo P., Shi X., Zhao Z., Dong X., Fei Z., Fu F. (2019). Comparison of electrical impedance tomography and intracranial pressure during dehydration treatment of cerebral edema. NeuroImage: Clinical.

[9] Sapuan I., Ain K., Suryanto A. (2017). Dual frequency electrical impedance tomography to obtain functional image. Journal of Physics: Conference Series.

[10] Ain K., Rahma O.N., Putra A.P., Syavinas N., Darmawan D., Sohal H. (2023). Bone fracture detection using electrical impedance tomography based on STEMlab Red Pitaya. Indonesian Journal of Electrical Engineering and Computer Science.

[11] Roh S., Koshima I., Mese T., Imai H., Aoki G., Kawano R., Yoshida S. (2023). Bioelectrical impedance analysis in patients with breast cancer-related lymphedema before and after lymphaticovenular anastomosis. Journal of Vascular Surgery: Venous and Lymphatic Disorders.

[12] Peixoto M., Moreno M.V., Khider N. (2021). Conception of a phantom in agar-agar gel with the same bio-impedance properties as human quadriceps. Sensors.

[13] Arnold M., Morgan E., Rumgay H., Mafra A., Singh D., Laversanne M., Vignat J., Gralow J.R., Cardoso F., Siesling S., Soerjomataram I. (2022). Current and future burden of breast cancer: Global statistics for 2020 and 2040. Breast..

[14] Adler A., Arnold J.H., Bayford R., Borsic A., Brown B., Dixon P., Faes T.J.C., Frerichs I., Gagnon H., Gärber Y., Grychtol B., Hahn G., Lionheart W.R.B., Malik A., Patterson R.P., Stocks J., Tizzard A., Weiler N., Wolf G.K. (2009). GREIT: A unified approach to 2D linear EIT reconstruction of lung images. Physiol. Meas..

[15] Harikumar R., Prabu R., Raghavan S. (2013). Electrical Impedance Tomography (EIT) and Its Medical Applications: A Review. International Journal of Soft Computing and Engineering (IJSCE).

[16] Badu-Peprah A., Adu-Sarkodie Y. (2018). Accuracy of clinical diagnosis. mammography and ultrasonography in preoperative assessment of breast cancer. Ghana Med. J..

[17] J.D. Bronzino, J.D. Enderle. (2012). Introduction To Biomedical Engineering Third Edition.

[18] Kalli S., Semine A., Cohen S., Naber S.P., Makim S.S., Bahl M. (2018). American Joint Committee on Cancer's Staging System for Breast Cancer. Eighth Edition: What the Radiologist Needs to Know. Radiographics..

[19] Bennett D. (2011). NaCl doping and the conductivity of agar phantoms. Materials Science and Engineering C.

[20] Bera T.K., Nagaraju J. (2009). 2009 IEEE International Advance Computing Conference.

[21] Bera T.K., Nagaraju J. (2012). Studying the resistivity imaging of chicken tissue phantoms with different current patterns in Electrical Impedance Tomography (EIT). Measurement: Journal of the InternationalMeasurementConfederation.

[22] Fraser C.J. (1994). Mechanical Engineer's Reference Book.

[23] Gagnon H., Cousineau M., Adler A., Hartinger A.E. (2010). A resistive mesh phantom for assessing the performance of EIT systems. IEEE Transactions on Biomedical Engineering.

[24] Khan T.A., Ling S.H. (2019). Algorithms.

[25] Mahnam A., Yazdanian H., Mosayebi Samani M. (2016). Comprehensive study of Howland circuit with non-ideal components to design high performance current pumps. Measurement: Journal of the International Measurement Confederation.

[26] Mansouri S., Alhadidi T., Azouz M.Ben (2020). Breast cancer detection using low-frequency bioimpedance device. Breast Cancer: Targets and Therapy.

[27] Miranda Mercado D.A., Godoy Alarcón E.V., V-Niño E.D (2024). Time evolution of electrical impedance spectra of Staphylococcus aureus and Escherichia coli bacteria. Bioelectrochemistry..

[28] Qiao, G., Wang, W., Wang, L., He, Y., Bramer, B., & Al-Akaidi, M. (2007). Investigation of biological phantom for 2D and 3D breast EIT images (Vol. 17). www.springerlink.com

[29] Sadleir R.J., Sajib S.Z.K., Kim H.J., Kwon O.I., Woo E.J. (2013). Simulations and phantom evaluations of magnetic resonance electrical impedance tomography (MREIT) for breast cancer detection. Journal of Magnetic Resonance.

[30] Soria, D.I. (2008). Implementation of an Electrical Bioimpedance Monitoring System and a Tool for Bioimpedance Vector Analysis.

[31] Wang N., Li Y., Zhao P.F., Huang L., Wang Z.Y. (2023). Fast electrical impedance tomography based on sparse Bayesian learning[Formula presented]. Appl. Soft. Comput..

[32] Widiana I.K., Irawan H. (2020). Clinical and Subtypes of Breast Cancer in Indonesia. Asian Pacific Journal of Cancer Care.

[33] Yang D., Gu C., Gu Y., Zhang X., Ge D., Zhang Y., Wang N., Zheng X., Wang H., Yang L., Chen S., Xie P., Chen D., Yu J., Sun J., Bai C. (2022). Electrical Impedance Analysis for Lung Cancer: A Prospective. Multicenter. Blind Validation Study. Front. Oncol..

[34] Yang L., Gao Z., Cao X., Fu F., Möller K., Frerichs I., Dai M., Zhao Z. (2023). The influence of gravity on electrical impedance tomography measurements during upper body position change. Heliyon..

[35] Zarkasi, A., & Santoso, D.R. (2017). Teknik Pencitraan 2D Distribusi Impedansi Listrik pada Zat Cair dengan Metode Berpasangan dan Menggunakan Software EIDORS.

[36] Zhang Y., Chen S., Sun X., Jing H., Zhou X. (2023). Electrochimica Acta.

[37] Ribeiro D.E., de Freitas Barbosa V.A., de Lima C.L., de Souza R.E., dos Santos W.P. (2020). Biomedical Computing For Breast Cancer Detection and Diagnosis.

[38] Holland S.S. (2020). 2020 IEEE International Symposium on Antennas and Propagation and North American Radio Science Meeting, IEEECONF 2020 - Proceedings.

